# The Rare Cuboid-Navicular Coalition Presenting as Chronic Foot Pain

**DOI:** 10.1155/2015/625285

**Published:** 2015-01-26

**Authors:** Omer Awan, James Allen Graham

**Affiliations:** ^1^Radiology, Geisel School of Medicine, One Medical Center Drive, Lebanon, NH 03756, USA; ^2^Dartmouth-Hitchcock Medical Center, One Medical Center Drive, Lebanon, NH 03756, USA; ^3^Radiology and Imaging Informatics, University of Maryland School of Medicine, 22 South Greene Street, Baltimore, MD 21201, USA

## Abstract

Tarsal coalitions are relatively rare diagnoses affecting adolescent patients that typically present with progressive foot pain. Cuboid-navicular coalition, a type of tarsal coalition, is extremely rare with less than 10 reported cases to date. Most prevailing theories reported have described this specific type of coalition as asymptomatic except at specific moments of stress and exercise. The purpose in presenting this case is to demonstrate that cuboid-navicular coalition can be associated with chronic unremitting pain, as in our patient. We present a case of cuboid-navicular fibrocartilaginous coalition in an adolescent patient presenting with chronic foot pain. Furthermore, from an imaging standpoint, radiographic findings are often subtle and radiologists cannot rely on indirect signs such as talar beak in clinching the diagnosis of cuboid-navicular coalition. Instead, abnormal articulation between the cuboid and navicular must be sought.

## 1. Introduction

Tarsal coalition presents in 1% of the population, with over 90% resulting from talocalcaneal or calcaneonavicular coalition [1–3]. Less than 1% of all coalitions have been reported to occur between the cuboid and the navicular, with less than 10 reported cases to date, none in the radiology literature [4, 5]. Classically, patients are asymptomatic but become symptomatic in adolescence as the coalition becomes more ossified. As much of the current literature points out, the two most common coalitions (talocalcaneal and calcaneonavicular) present as a painful foot in adolescence that can be exacerbated with walking, activity, or any exercise. However, current ideologies regarding cuboid-navicular coalitions elucidate the belief that this particular type of coalition is usually asymptomatic except at specific moments where activity or exercise can result in pain or peroneal spastic flatfoot [5–7].

The purpose of this paper is to introduce the reader to a case where the rare cuboid-navicular coalition resulted in chronic unremitting pain in the foot that was not predominantly asymptomatic. Furthermore, from an imaging perspective, radiologists often rely upon indirect signs of tarsal coalition on radiography such as the talar beak, which manifests as added bone formation along the dorsum of the talus secondary to abnormal motion of the talonavicular joint. Talar beak may be seen with talocalcaneal coalitions and less commonly with calcaneonavicular and other rarer coalitions [2, 8]. As our case will show, a radiologist cannot always rely on indirect signs such as the talar beak to make the diagnosis of a tarsal coalition.

## 2. Case Report

A 17-year-old otherwise healthy male with noncontributory past medical history presented to his orthopedic surgeon for evaluation of chronic unremitting right foot pain that had been bothering him for six months. He finally decided to visit his orthopedic surgeon after noting swelling along the medial aspect of his foot while playing lacrosse. The patient graded the pain as an 8/10 with nothing making the pain better or worse. No inciting event could be recalled by the patient that started the pain six months earlier. On physical exam, the patient was tender over the tarsonavicular region without any appreciable swelling. Range of motion around the foot was normal and the patient could bear weight. Blood results such as complete blood count, erythrocyte sedimentation rate, and C-reactive protein values were all within normal limits.

**Figure 1:**
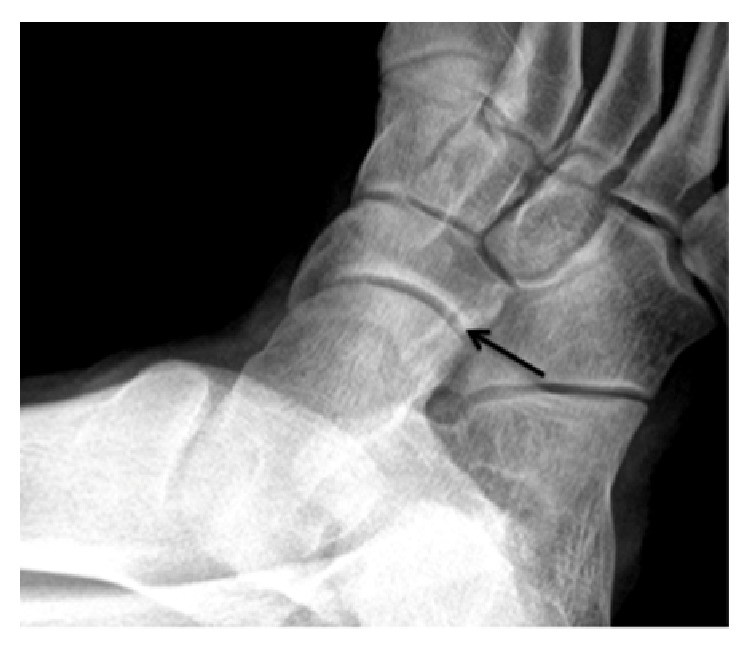
Magnified oblique radiograph of the right foot in a 17-year-old male better delineates the abnormal articulation between the cuboid (arrow) and the navicular.

**Figure 2:**
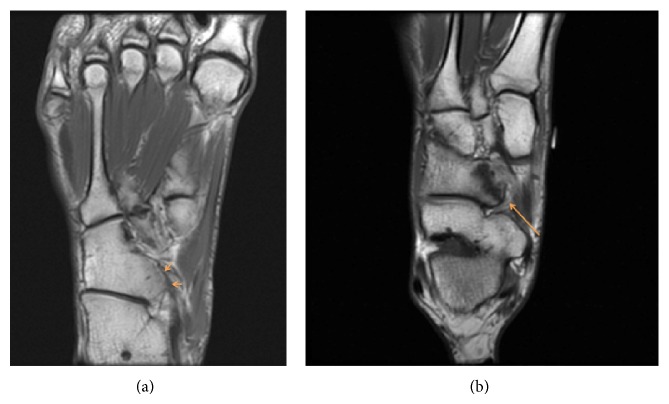
(a)-(b) Coronal T1 weighted unenhanced MR images of the right foot in a 17-year-old male show an elongated posterior medial process of the cuboid (arrows, (a)) that articulates abnormally with the plantar lateral aspect of the navicular (arrow, (b)), consistent with cuboid-navicular coalition.

**Figure 3:**
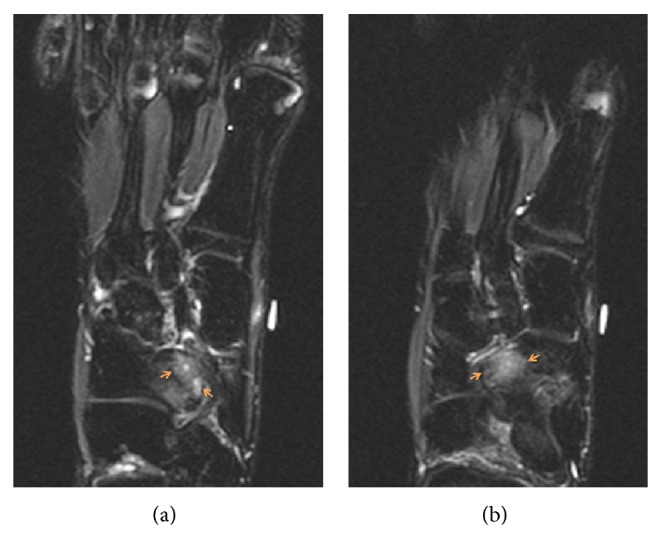
(a)-(b) Coronal short tau inversion recovery (STIR) unenhanced MR images of the right foot in a 17-year-old male with cuboid-navicular coalition demonstrate marrow edema and cystic change in the cuboid (arrows, (a)) as well as bone marrow edema in the navicular (arrows, (b)).

**Figure 4:**
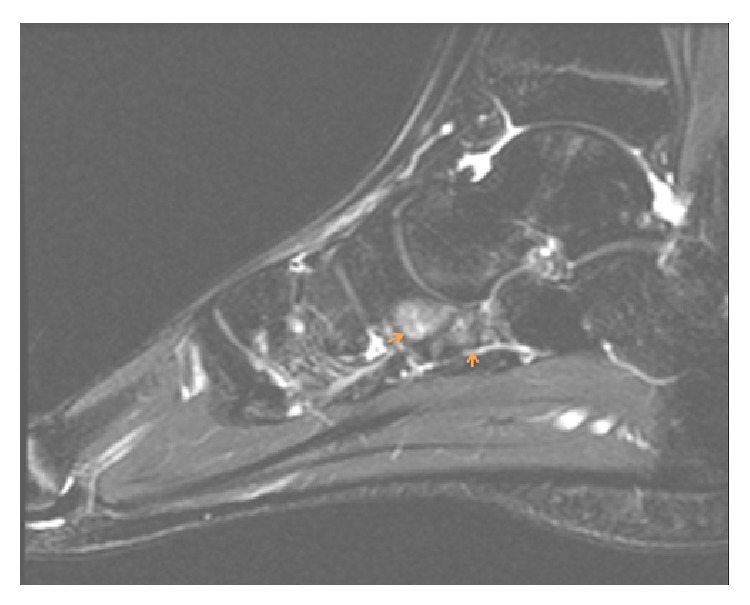
Sagittal STIR unenhanced MR image of the right foot in a 17-year-old male with cuboid-navicular coalition reveals abnormal articulation between the cuboid and navicular as well as marrow edema along both sides of the coalition (arrows).

The orthopedic surgeon decided to order radiographs of the foot that initially were read as negative for fracture or any significant abnormality by the radiologist. In retrospect, the posterior medial aspect of the cuboid articulated abnormally with the plantar lateral aspect of the navicular (Figure 1). Importantly, no talar beak was visualized on radiography. The orthopedic surgeon then ordered magnetic resonance imaging (MRI) of the right foot without intravenous contrast to further elucidate the etiology of the patient's symptoms. MRI revealed abnormal articulation between the cuboid and navicular as well as marrow edema on both sides of the coalition with cystic change along the cuboid (Figures 2(a), 2(b), 3(a), 3(b), and 4), consistent with fibrocartilaginous coalition as no osseous connection was seen between the cuboid and navicular. The patient was treated conservatively with physical therapy that helped for three months and is scheduled to receive a cortisone injection into the coalition if necessary for further alleviation of symptoms. However, to date, the patient has not reported pain recurrence and cortisone injection has been deferred until the patient presents again with pain. The orthopedic surgeon never ordered computed tomography (CT) examination since surgical planning was not needed in this case. No further imaging has been performed on this patient after his baseline MRI foot examination.

## 3. Discussion

Tarsal coalitions represent abnormal bridging between tarsal bones and can be osseous, cartilaginous, or fibrous. They develop secondary to failure of differentiation and segmentation of the primitive mesenchyme in the first stages of development [9]. There can be a genetic component with autosomal dominance with variable penetrance, and patients can also have clinodactyly, hereditary symphalangism, and ball-and-socket ankle joint with a great toe shorter than the second toe [5]. Males are slightly more likely to exhibit tarsal coalition than females and patients classically develop progressive pain and stiffness in the foot with decreased hindfoot and midfoot motion on clinical exam [10].

The two most common tarsal coalitions can be readily diagnosed with conventional radiography, namely, by noting the “C” sign, or sclerosis in an inverted “C” shape of the calcaneus on the lateral view in the case of talocalcaneal coalition as well as the “anteater” sign or elongated anterior process of the calcaneus in the case of a calcaneonavicular coalition [1, 2]. Direct visualization of the abnormal articulation proves to be the most reliable method of confidently diagnosing a tarsal coalition. Radiologists are also taught to look for indirect signs of tarsal coalition such as the talar beak that again results from abnormal weight bearing mechanics at the talocalcaneal joint from various forms of tarsal coalitions [1, 11].

Cuboid-navicular coalition remains an extremely rare form of tarsal coalition [12, 13]. For reasons not well known to this day, much of the literature including reports from Garcia-Mata, Williamson and Torode, and Waugh emphasizes the notion that this particular form of tarsal coalition is essentially asymptomatic and rarely becomes symptomatic at specific times of activity and stress [5–7]. Our case demonstrates precisely the opposite scenario where cuboid-navicular coalition was associated with chronic unremitting pain at rest that eventually did become exacerbated to some degree with lacrosse playing.

Careful attention to detail is necessary in diagnosing cuboid-navicular coalition on conventional radiography. The cuboid normally articulates with the fourth and fifth metatarsals, the lateral cuneiform, the calcaneus, and the navicular. Often, the only clue to the diagnosis of cuboid-navicular coalition on radiography may be an abnormal articulation between the posterior medial cuboid and plantar lateral navicular with marked superimposition of the cuboid over the navicular bone, as in our case. A talar beak will never be present in this type of coalition as there is no alteration in the weight bearing mechanics of the talonavicular joint in a cuboid-navicular coalition [14]. Thus, the lack of a talar beak cannot be confidently used to exclude the presence of a tarsal coalition. While a talar beak is not always present in various tarsal coalitions, radiologists often search for this finding in corroborating a diagnosis of tarsal coalition. The coalition in our case was initially missed on conventional radiography as no indirect signs of coalition were present.

Symptomatic coalitions may be treated conservatively with nonsteroidal anti-inflammatory drugs, casting, steroid injection, and orthotics or can be treated surgically with excision. More recently, symptomatic cuboid-navicular coalitions have been treated through the resection and interposition of an adipose graft in a handful of patients [15]. The patient in our case benefitted from physical therapy alone and may pursue cortisone injection into the coalition for further relief of symptoms.

In summary, we present a rare case of cuboid-navicular coalition that resulted in marked discomfort and chronic pain for our patient. Our purpose in presenting this case was to debunk the belief that this specific type of coalition is asymptomatic except at specific moments of stress as our patient had chronic pain even at rest. Furthermore, radiographic findings of this coalition are subtle and often missed, as in our example. Indirect signs such as talar beak cannot always be used to reliably exclude the diagnosis of tarsal coalition and direct visualization of the abnormal articulation between tarsal bones should be sought to confidently clinch the diagnosis.
